# Low Prevalence of Transmitted Drug Resistance in Patients Newly Diagnosed with HIV-1 Infection in Sweden 2003–2010

**DOI:** 10.1371/journal.pone.0033484

**Published:** 2012-03-20

**Authors:** Annika Karlsson, Per Björkman, Göran Bratt, Håkan Ekvall, Magnus Gisslén, Anders Sönnerborg, Mattias Mild, Jan Albert

**Affiliations:** 1 Department of Laboratory Medicine, Karolinska Institute, Stockholm, Sweden; 2 Department of Clinical Sciences, Malmö Infectious Disease Research Unit, Malmö University Hospital, Lund University, Lund, Sweden; 3 Venhälsan, Stockholm South General Hospital, Stockholm, Sweden; 4 Department of Infectious Diseases, Sundsvall Hospital, Sundsvall, Sweden; 5 Department of Infectious Diseases, the Sahlgrenska Academy at the University of Gothenburg, Gothenburg, Sweden; 6 Department of Infectious Diseases, Karolinska University Hospital, Stockholm, Sweden; 7 Department of Clinical Microbiology, Karolinska University Hospital, Stockholm, Sweden; 8 Department of Microbiology, Tumor and Cell Biology, Karolinska Institute, Stockholm, Sweden; 9 Swedish Institute for Communicable Diseases, Stockholm, Sweden; Centro Nacional de Microbiología - Instituto de Salud Carlos III, Spain

## Abstract

Transmitted drug resistance (TDR) is a clinical and epidemiological problem because it may contribute to failure of antiretroviral treatment. The prevalence of TDR varies geographically, and its prevalence in Sweden during the last decade has not been reported. Plasma samples from 1,463 patients newly diagnosed with HIV-1 infection between 2003 and 2010, representing 44% of all patients diagnosed in Sweden during this period, were analyzed using the WHO 2009 list of mutations for surveillance of TDR. Maximum likelihood phylogenetic analyses were used to determine genetic subtype and to investigate the relatedness of the sequences. Eighty-two patients showed evidence of TDR, representing a prevalence of 5.6% (95% CI: 4.5%–6.9%) without any significant time trends or differences between patients infected in Sweden or abroad. Multivariable logistic regression showed that TDR was positively associated with men who have sex with men (MSM) and subtype B infection and negatively associated with CD4 cell counts. Among patients with TDR, 54 (68%) had single resistance mutations, whereas five patients had multi-drug resistant HIV-1. Phylogenetic analyses identified nine significantly supported clusters involving 29 of the patients with TDR, including 23 of 42 (55%) of the patients with TDR acquired in Sweden. One cluster contained 18 viruses with a M41L resistance mutation, which had spread among MSM in Stockholm over a period of at least 16 years (1994–2010). Another cluster, which contained the five multidrug resistant viruses, also involved MSM from Stockholm. The prevalence of TDR in Sweden 2003–2010 was lower than in many other European countries. TDR was concentrated among MSM, where clustering of TDR strains was observed, which highlights the need for continued and improved measures for targeted interventions.

## Introduction

Human immunodeficiency virus type 1 (HIV-1) is characterized by a rapid rate of evolution and is therefore prone to accumulate mutations that confer resistance to antiretroviral drugs during suboptimal antiretroviral therapy (ART). Viruses with resistance mutations may be transmitted and such transmitted drug resistance (TDR) is an important clinical and epidemiological problem, because it can contribute to failure of first-line ART [1]. For this reason the World Health Organization (WHO) recommends that countries that have installed ART programs should establish sentinel surveillance systems of TDR and provide evidence-based recommendations for prevention of HIV drug resistance [2], [3]. Furthermore, Swedish and international treatment guidelines recommend that resistance testing should be performed in newly diagnosed patients [4]–[6]. The reported prevalence of TDR in more recent, large studies from different European countries and the United States (US) range from 6.1% in Denmark to 14.6% in the U.S. [7]–[12]. Furthermore, the pan-European SPREAD study reported a TDR prevalence of 8.4% in Europe in 2002–2005 [13], [14].

Sweden, with a population of approximately 10 million inhabitants, has a low prevalence and incidence of HIV-1. At the end of 2010 there had been a cumulative number of 9,400 diagnosed cases and approximately 5,300 persons were estimated to be living with HIV-1 in the country (www.smi.se). Among 3,257 patients with known transmission route diagnosed in 2000–2009, 61% were heterosexually infected, 29% were men who have sex with men (MSM) and 10% were intravenous drug users (IDUs). The proportion of patients infected abroad was 86%, 44% and 25% in these three transmission groups, respectively. Immigrants from high-prevalence countries constituted 60% of the patients with heterosexually acquired HIV-1 infection (ecdc.europa.eu). ART has been universally available in Sweden since zidovudine was introduced in 1986 and is prescribed by specialists in infectious diseases or equivalent according to national HIV treatment guidelines [4] (updated version available in Swedish at www.rav.nu). Despite the long use of ART in Sweden there is incomplete knowledge about TDR and there have only been three previous studies which were relatively small and investigated patients diagnosed before 2003 [15]–[17]. The present study was designed to prospectively and longitudinally monitor TDR in Sweden using representative sampling. We report results for 1463 patients diagnosed between 2003 and 2010, which represents 44% of all patients diagnosed in Sweden during this period. The prevalence of TDR was relatively low, 5.6%.

## Materials and Methods

### Patients

Patients were recruited from January 2003 until June 2010. The five clinical centers for HIV care in the three largest Swedish cities (Stockholm, Gothenburg and Malmö) took part in the study from the start. Fifteen additional HIV centers were added from 2005 so that 20 of the 29 centers in Sweden eventually took part in the study. The study was coordinated with the EU project SPREAD (Strategy to Control SPREAD of HIV drug resistance) (www.esar-society.eu), which is a multinational, multicentre surveillance program aimed at determining the prevalence of TDR in Europe. The study protocols and data collection forms of SPREAD were used with a few modifications. Patient data included age, gender, country of origin, suspected country of acquisition of infection, clinical staging, transmission route, last negative and first positive HIV test, laboratory evidence of primary HIV-1 infection. Laboratory documented primary HIV-1 infection was defined as an acute seroconversion syndrome confirmed by: a) HIV-1 antibody seroconversion, or b) HIV-1 antibody negativity+positive HIV-1 RNA PCR, or c) HIV-1 antibody negativity+positive HIV-1 DNA PCR, or d) HIV-1 antibody negativity+positive HIV-1 antigen test; in all cases later re-confirmed by a full HIV-1 antibody seroconversion. Patients originating from high-prevalence countries (defined as countries with a national HIV prevalence above 1% according to the 2002 UNAIDS estimates) were considered to have acquired HIV-1 infection heterosexually unless specifically reported as being MSM or IDUs. The inclusion criteria were: 1) HIV-1 infection newly diagnosed in Sweden during the study period; 2) Sample for resistance testing obtained within 180 days following diagnosis; 3) No known exposure to ART;. Exclusion criteria were: 1) HIV-2 infection. 2) HIV-1 infection previously diagnosed outside of Sweden.

Informed written or oral consent was obtained from all adult participants and from the next of kin, caregivers or guardians on the behalf of the minors/children participants. The research was conducted according to the Declaration of Helsinki and was approved by the Regional Medical Ethics Board in Stockholm, Sweden (Dnr 02-367, 04-797 and 2007/1533) that had permitted the use of oral consent, which was documented in the patient records, to minimize the risk of selection biases due to patient drop-out because some ethnic groups of participants were known to be willing to take part in the study, but reluctant to provide written consent.

### Measurements of plasma HIV-1 RNA levels and CD4 counts

Plasma HIV-1 RNA levels were measured using the Cobas AmpliPrep sample preparation system followed by analysis using the Cobas Amplicor HIV-1 monitor version 1.5 or the Cobas TaqMan HIV-1 v1.0 or v2.0 (Roche Molecular Systems, Basel, Switzerland). CD4+ T-lymphocyte (CD4) cells were enumerated using flow cytometry.

### Genotypic HIV-1 resistance testing

Genotypic HIV-1 resistance testing for 1368 of the samples was performed at the Swedish Institute for Infectious Disease Control using a published in-house method that targets amino acids 1–99 in the protease and 1–253 in the reverse transcriptase [18]. The test has undergone regular quality control within ENVA genotyping proficiency programme [19]. Resistance testing was performed on plasma samples that had been stored at −70°C until testing. If testing of plasma was unsuccessful, we attempted analysis on HIV-1 DNA from peripheral blood mononuclear cells (PBMCs) if available. For 12 patients we used sequences obtained from PBMCs, 7 of these patients had plasma HIV-1 RNA levels lower than 1000 copies/mL. For 83 patients we obtained sequences from routine resistance testing performed at the Karolinska University Hospital (n = 12) or the Sahlgrenska University Hospital (n = 71). To qualify for the subsequent analyses the sequences had to include amino acids 15–90 in the protease and amino acids 41–219 in the reverse transcriptase, but a majority of the sequences covered the entire amplicon length of the in-house resistance test.

The sequences were assembled and edited using the Sequencher™ software (Gene Codes Corporation, Ann Arbor, MI, US). TDR was identified using the WHO 2009 list of mutations for surveillance of TDR [20] as implemented in the Calibrated Population Resistance tool (v5.0 beta) [21] available at the Stanford HIV Drug Resistance Database (hivdb.stanford.edu). The following resistance mutations were scored: to nucleoside reverse transcriptase inhibitors (NRTIs): M41L, K65R, D67N/G/E, T69D/insertion, K70R/E, L74V/I, V75M/T/A/S, F77L, Y115F, F116Y, Q151M, M184V/I, L210W, T215Y/F/I/S/C/D/V/E, K219Q/EN/R; to non-nucleoside reverse transcriptase inhibitors (NNRTIs): L100I, K101E/P, K103N/S, V106A/M, V179F, Y181C/I/V, Y188C/L/H, G190A/S/E, P225H, M230L; and to protease inhibitors (PIs): L23I, L24I, D30N, V32I, M46I/L, G48V/M, I50L/V, F53L/Y, I54V/L/M/A/T/S, G73S/T/C/A, L76V, V82A/T/F/S/C/M/L, N83D, I84V/A/C, N88D/S, L90M. The susceptibility of the viruses to antiretroviral drugs was predicted using the Stanford HIVdb, ANRS and Rega algorithms as implemented in the HIValg tool also available at the Stanford HIV Drug Resistance Database.

### Subtype determinations and phylogenetic tree analyses

To determine the HIV-1 genetic subtype of the viruses, the *pol* sequences were manually aligned with the subtype reference sequence dataset from the Los Alamos HIV Sequence Database (www.hiv.lanl.gov) using BioEdit [22]. Because resistance mutations might lead to homoplasy (convergent evolution) all 23 codons with resistance mutations in the dataset were removed. Subtype determination was done by maximum likelihood (ML) phylogenetic trees using PhyML [23] with the GTR+I+G substitution model, which was the best fitted model according to Findmodel (www.hiv.lanl.gov). Problematic sequences were further investigated using the Rega HIV-1 Subtyping Tool (version 2.0) (http://regaweb.med.kuleuven.be), HIV BLAST analyses (hiv.lanl.gov), and Simplot analyses (http://sray.med.som.jhmi.edu/SCRoftware/simplot). ML phylogenetic trees were also used to study detailed evolutionary relationships within subtype B. In these analyses we included previously published sequences from 194 MSM who were newly diagnosed with HIV-1 infection in Stockholm, Sweden in 1992–2002 [17]. Sequence clusters were defined as two or more sequences that were significantly separated from the rest of the tree by an approximate likelihood ration test (aLRT) value>0.95 in PhyML [24], but bootstrap analyses were also performed using neighbor-joining phylogenetic trees that were constructed with the maximum composite likelihood substitution model in Mega v4.1 [25]. For similar scientific and ethical reasons as explained in [26], [27], only a proportion (approximately 15%) of the anonymized sequences is accessible via GenBank (accession numbers, JQ698667–JQ698874). In brief, the sequences analyzed in the present study constitute a dataset that is very representative of an entire country and thereby, in principle, allow for the reconstruction of entire transmission networks. Inappropriate use of the data could thereby endanger the privacy of the patients, which is especially problematic because HIV-1 sequences frequently have been used in court cases. Furthermore and from a scientific point of view, the consequences of open and uncontrolled access to such densely sampled sequences could jeopardize the future publication (and, thus, the investigation) of similarly complete datasets and could thereby be counterproductive even from an “open-access” perspective [27]. However, the entire dataset can be used for well-defined projects that have passed Swedish ethical clearance and are in accordance with the guidelines of the Swedish cohort, if a corresponding project proposal is approved by the scientific board.

### Statistical analyses

The 95% confidence interval (95% CI) of the prevalence of TDR was calculated using the binomial distribution and the exact method. The Chi-square and Mann-Whitney U tests were used as appropriate. Univariable and multivariable logistic regression analyses were used to estimate odds ratios with 95% CI for the association between TDR status and different factors. Statistical analyses were carried out using Statistica v10 and Stata v 8.2.

## Results

### General characteristics of the study subjects

A total of 1491 patients met the inclusion criteria. Most of study subjects (n = 1009) were recruited from Stockholm; of the remaining patients 147 were from Gothenburg, 122 from Malmö and 213 from the rest of the country. Six eligible patients were excluded prior to data analysis because the sequence did not include all relevant resistance positions and 22 patients were excluded because resistance testing was unsuccessful. The latter patients had a median plasma HIV-1 RNA level of 500 copies/mL and 14 patients had <1000 copies/mL. Thus, the analyses included 1463 patients, of whom 291 (20%) were defined as having a recent infection based on a laboratory documented primary HIV-1 infection or a negative HIV-1 serology <1 year prior to diagnosis. The remaining patients had HIV-1 infections of unknown duration. The median time between diagnosis and sampling for resistance testing was 11 days (range 0–180 days).

The general characteristics of the study subjects are shown in Table 1. Approximately 70% of the study subjects were men and 30% were females. The median age of all study subjects was 38 years and men were significantly older than women (median age 40 years vs. 33 years; p<0.001, Mann-Whitney U-test). The most frequently reported transmission routes were: heterosexual (51%, with 31% originating from high-prevalence countries), MSM (37%) and IDU (9%). A high proportion of the patients (832 of 1463; 57%) were reported to have been infected abroad. The most frequently reported countries of infection were: Sweden (42%), Thailand (12.3%), Ethiopia (3.3%), Eritrea (2.7%), Kenya (2.4%), Spain (1.9%), and Somalia (1.7%). The distribution of countries of origin was quite similar to the countries of infection (data not shown).

**Table 1:** Characteristics of patients with and without transmitted drug resistance (TDR).

Characteristics	Total	Patients with TDR (%)		Patients without TDR (%)		OR univariable (95% CI)		P-value
Patients	1463	82	(5.6%)	1381	(94.4%)			
Sex [n]								
Female	440	13	(3.0%)	427	(97.0%)	1		
Male	1023	69	(6.7%)	954	(93.3%)	2.38	(1.3–4.3)	0.005
Age [median (range)]	38	39	(1–83)	37	(13–64)			0.27
Year of diagnosis [n]						1.05	(0.95–1.2)	0.34
2003	126	7	(5.6%)	119	(94.4%)			
2004	153	8	(5.2%)	145	(94.8%)			
2005	155	11	(7.1%)	144	(92.9%)			
2006	192	4	(2.1%)	188	(97.9%)			
2007	239	10	(4.2%)	229	(95.8%)			
2008	221	16	(7.2%)	205	(92.8%)			
2009	241	18	(7.5%)	223	(92.5%)			
2010	136	8	(5.9%)	128	(94.1%)			
Route of transmission [n]								
Homosexual/bisexual	536	51	(9.5%)	485	(90.5%)	1		
Intravenous drug use	136	4	(2.9%)	132	(97.1%)	0.29	(0.10–0.81)	0.019
Heterosexual	752	25	(3.3%)	727	(96.7%)			
High-prevalence country	453	13	(2.9%)	440	(97.1%)	0.28	(0.15–0.52)	>0.001
Low-prevalence country	299	12	(4.2%)	287	(96.0%)	0.40	(0.21–0.76)	0.005
Mother-to-child	9	1	(11.1%)	8	(88.9%)			
Other/Unknown	30	1	(3.3%)	29	(96.7%)			
Region of infection [n]								
Sweden	631	42	(6.7%)	589	(93.3%)	1		
Europe, except Sweden	128	12	(9.4%)	116	(90.6%)	1.45	(0.74–2.8)	0.28
Sub-Saharan Africa	336	10	(3.0%)	326	(97.0%)	0.43	(0.21–0.87)	0.019
Asia	216	9	(4.2%)	207	(95.8%)	0.61	(0.29–1.3)	0.19
Americas	51	3	(5.9%)	48	(94.1%)	0.88	(0.26–2.9)	0.83
Other/Missing data	101	6	(5.9%)	95	(94.1%)			
HIV-1 subtype [n]								
Subtype B	605	55	(9.1%)	550	(90.9%)	1		
Subtype A	130	2	(1.5%)	128	(98.5%)	0.15	(0.04–0.65)	0.011
Subtype C	224	5	(2.2%)	219	(97.8%)	0.23	(0.09–0.57)	0.002
CRF01_AE	273	10	(3.7%)	263	(96.3%)	0.38	(0.19–0.76)	0.006
CRF02_AG	114	2	(1.8%)	112	(98.2%)	0.18	(0.04–0.76)	0.020
Subtype D	25	0	(0.0%)	25	(100%)			
Other	92	8	(8.7%)	84	(91.3%)			
Duration of infection [n]								
Undefined	1172	67	(5.7%)	1105	(94.3%)	1		
<1 year	291	15	(5.2%)	276	(94.8%)	0.89	(0.50–1.6)	0.70
CD4+ T-cell counts (cells/µl )								
median (range)	320	279	(0–1480)	325	(0–2030)			0.34
Plasma HIV-1 RNA levels [log copies/ml								
median (range)]	4.8	4.8	(2.3–6.8)	4.8	(<1.6–7.6)			0.99

The characteristics of the study subjects were compared to national HIV surveillance data (www.smi.se) (Table S1). Overall, we sampled around 44% of all patients diagnosed in Sweden in 2003–2009 and in addition 136 patients who were diagnosed in the first half of 2010. The distribution of the study population was reasonably well matched with that of all diagnosed patients. However, MSM were somewhat over-represented in the study population (36% vs. 24%), but it should be noted that data on the route of transmission were not available for 16% of the patients in the national data. In agreement with this, we observed an over-representation of men and individuals infected in Sweden in the study population.

### Genetic HIV-1 subtypes

The genetic subtype of the sequenced *pol* gene fragment was subtype B for 41% of the patients, circulating recombinant form 01_AE (CRF01_AE) 19%, subtype C 15%, subtype A 9%, CRF02_AG 8% and subtype D 2%. Remaining patients (6%) had virus that was classified as other subtypes (F and G), circulating recombinant forms (CRFs) (CRF03_AB, CRF06_cpx, CRF07_BC, CRF09_cpx, CRF10_CD, CRF11_cpx, CRF12_BF, CRF13_cpx, CRF20_BG, CRF24_BG, CRF33_01B, CRF34_01B, CRF35_AD, CRF49_cpx), unique recombinant forms or unclassifiable (data not shown). The large proportion of CRF01_AE infections was linked to travel to and immigration from Thailand where CRF01_AE is common, but also to an outbreak of CRF01_AE infections among IDUs in Stockholm in 2007 [28]. In line with this, the proportion of subtype B infections decreased significantly over time among patients infected in Sweden (p<0.001), while the proportion of CRF01_AE infections increased significantly (p = 0.015).

### TDR over-represented in MSM and subtype B

Eighty-two of the 1463 study subjects had viruses with mutations indicative of TDR according to the WHO 2009 list of mutations for surveillance of transmitted drug resistance [20] (Table 1). Thus, the prevalence of TDR was 5.6% (95% CI: 4.5%–6.9%) in this study population. The prevalence of TDR was significantly higher among men than in women (OR 2.38; 95% CI 1.30–4.34) (Table 1), which can be explained by the higher prevalence of TDR among MSM (9.5%) than in the other three main transmission groups.

The prevalence of TDR was slightly, but non-significantly, higher among patients infected in Sweden or abroad (42 of 631 [6.7%] vs. 40 of 832 [4.8%], p = 0.13; Chi square test). However, when patients infected abroad were broken down into subgroups, the prevalence of TDR was significantly lower in patients infected in Sub-Saharan Africa (3.3%, p = 0.019) and non-significantly higher among patients infected in the rest of Europe (9.4%, p = 0.28) as compared to patients infected in Sweden. There were no significant differences in prevalence of TDR among MSM infected in Sweden or abroad (8.8% vs 10.9%; p = 0.44, Chi square test) or among patients who were heterosexually infected in Sweden vs. other low prevalence countries (4.8% vs. 3.7%; p = 0.66 Chi square test). The study was not powered to carry out such comparison for the remaining transmission groups. The prevalence of TDR did not differ between patients attending care in Stockholm, Gothenburg, Malmö or other study sites. TDR was more common among patients infected with subtype B (9.1%) than among patients infected with other subtypes (3.1%) (p = 0.0025, Chi square test), whereas differences between other subtypes were non-significant (data not shown). Patients with recent infections (<1 year) and patients with unknown duration of infection had similar prevalence of TDR (15 of 291 [5.2%] vs. 67 of 1172 [5.7%]; p = 0.71, Chi square test). Among the 15 patients with recent infections and TDR, 10 were MSM infected in Sweden indicating that viruses with TDR mutations were being transmitted among MSM in Sweden during the study period. Patients with and without TDR had similar median CD4 cell counts and plasma HIV-1 RNA levels.

Based on the univariable statistical analyses, several multivariable logistic regression models were explored. The final model is shown in Table 2, which showed that TDR was positively associated with the MSM transmission route, subtype B infection and negatively associated with log transformed CD4 cell counts.

**Table 2:** Multivariable model showing factors that were significantly associated with TDR.

Variable	Odds ratio	95% CI	P-value
MSM	2.15	1.14–4.07	0.018
Subtype B	1.96	1.03–3.73	0.041
Ln CD4 counts	0.81	0.69–0.96	0.013

### Trend towards increasing TDR among patients infected in high-prevalence countries

The prevalence of TDR showed relatively high variation over the study period; from 2.1% in 2006 to 7.5% in 2009 (Table 1), but there was no clear trend over time even if infection route was included as a confounder to adjust for the higher prevalence of TDR among MSM (p = 0.32, logistic regression). When time trends were investigated for individual transmission groups, we observed a non-significant trend towards an increasing prevalence of TDR among patients from high-prevalence countries (p = 0.071; logistic regression), but this should be interpreted with caution because there were only 13 such patients with TDR. In the three remaining major transmission groups, i.e. IDUs, MSM, heterosexual transmission in low-endemic countries, there were no significant changes in TDR over time.

### A majority of patients with TDR had singleton resistance mutations

A majority of the patients with TDR (56 of 82; 68%) had virus with single drug resistance mutations (Table 3). Of these singleton mutations, 35 were NRTI-related, 16 were NNRTI-related and 5 were PI-related. The M41L mutation represented almost half (16 of 34) of the NRTI-related singleton mutations and the K103N mutation represented two-thirds (10 of 15) of the NNRTI-related singleton mutations. As shown in Table 3, 80% (28 of 35) of the patients with single mutations associated with NRTI resistance were predicted to be fully susceptible to all NRTIs according to the Rega algorithm. Similarly, all five patients with single PI-related mutations were predicted to be fully susceptible to all PIs. In contrast, 14 of 16 patients with NNRTI-related singleton mutations were predicted to have high-level resistance to efavirenz and nevirapine, but resistance to etravirin was uncommon. Drug susceptibility prediction using the Stanford or ANRS algorithms gave similar, but not identical, results (data not shown).

**Table 3:** Susceptibility of HIV-1 strains with single transmitted drug resistance mutations.

Patient id	n	Mutation^1^	Predicted susceptibility to						
		NRTI related	AZT	D4T	FTC	3TC	ABC	DDI	TDF
1–16^2^	16	M41L	S	S	S	S	S	S	S
17	1	D67N	S	S	S	S	S	S	S
18	1	K70E	S	S	I	I	I	I	I
19–23	5	F77L	S	S	S	S	S	S	S
24	1	M184V	S	S	R	R	S	S	S
25–29^3^	5	T215C/S	I	I	S	S	S	S	S
30–35	6	K219N/Q/R	S	S	S	S	S	S	S

		NNRTI related	EFV	NVP	ETR
36–37	2	K101E	S	S	S
38–47^4^	10	K103N	R	R	S
48–49^5^	3	Y181C	R	R	I
51	1	G190AG	R	R	S

		PI related	ATV/r	DRV/r	FPV/r	IDV/r	LPV/r	SQV/r	TPV/r
52–54	3	M46L	S	S	S	S	S	S	S
55	1	I54IL	S	S	S	S	S	S	S
56	1	I85V	S	S	S	S	S	S	S

Susceptibility was predicted using the Rega resistance interpretation algorithm (V6.4.1) (ref). NNRTI, non-nucleoside reverse transcriptase inhibitor; NRTI, nucleoside reverse transcriptase inhibitor; PI, protease inhibitor;

1) In eight patients the single resistance mutation was present as a polymorphism together with wild-type virus;

2) Ten patients in cluster no 4, two patients in cluster no. 5, one patient in cluster no. 2;

3) Two patients (with T215S) in cluster no. 6;

4) Two patients in cluster no. 7 and two patients in cluster no. 8;

5) Two patients in cluster no. 9.

Twenty-six patients had viruses with more than one TDR mutation (Table 4). A majority of these patients were MSM who had been infected in Sweden. Multidrug resistance (MDR) involving all three drug as well as the Q151M complex [29] was observed in five patients (patients 57–61). Dual class resistance was observed in five patients, whereas the remaining 17 patients had two or more TDR mutations belonging to a single drug class. A majority of the latter patients had thymidine analogue mutations (TAMs) [29].

**Table 4:** Characteristics and susceptibility of HIV strains with multiple transmitted drug resistance mutations.

							Resistance mutations			Number of fully active drugs		
Patient ID	Cluster no. (size)	Year of diagnosis	Transmission route	Country of infection	CD4 count	Subtype	NRTI	NNRTI	PI	NRTI (7)	NNRTI (3)	PI/r (7)
57	1 (5)	2003	MSM	Sweden	976	B	F77L, Y115F, F116Y, Q151M	K103N	L24I, M46I, I50V, I54V, V82A	3	1	1
58	1 (5)	2003	MSM	Sweden	287	B	K70R, F77L, F116Y, Q151M	K103N	M46I, I50V, I54V, V82A	3	1	1
59	1 (5)	2004	MSM	Sweden	877	B	F77L, F116Y, Q151M	K103N	I50V, I54V, V82A	3	1	3
60	1 (5)	2005	MSM	Sweden	351	B	F77L, Q151M	K103N	I50V, I54V	3	1	4
61	1 (5)	2010	MSM	Sweden	172	B	F77L, F116Y, Q151M	K103N	I50V	3	1	6
62		2009	MTCT	Thailand	80	CRF01_AE	M41L, D67DN, M184I, L210W, T215Y	K101E, Y181C, G190A		0	0	7
63		2009	MSM	USA	300	B	M41LM, L210LW, T215D			3	3	7
64	2 (4)	2004	MSM	Sweden	180	B	M41LM, L210LW, T215D			3	3	7
65		2003	MSM	Sweden	270	B	D67G, T215S, K219Q			5	3	7
66		2009	MSM	Turkey	645	B	M41L, T215CDGY	K103N		4	1	7
67		2007	MSM	Lithuania	565	F	M41L, D67N			5	3	7
68		2008	MSM	Sweden	340	B	M41L, T215D			5	3	7
69		2004	MSM	Sweden	9	B	M41L, T215S			5	3	7
70		2009	MSM	Sweden	570	B	M41L, T215S			5	3	7
71	3 (3)	2004	MSM	Spain	757	B	M41L, T215S			5	3	7
72		2008	MSM	Europe	260	B	M41L, T215S			5	3	7
73		2009	MSM	Denmark	50	B	M41LM, T215ST			5	3	7
74	4 (18)	2005	MSM	Sweden	298	B	M41L		M46LM	7	3	7
75		2008	HSX	Romania	50	F	D67DN, K219Q			5	3	7
76	2 (4)	2010	IDU	Sweden	20	B	L210W, T215D			5	3	7
77		2007	MSM			B	K219EK	Y181CY		7	0	7
78		2009	High prev	Thailand	154	CRF01_AE		K103KN, G190AG		7	1	7
79		2008	MSM	Sweden	682	B		K103N, P225H		7	1	7
80		2009	MSM		152	B		K103KN, P225HPSY		7	1	7
81		2003	MSM	Russia	130	B		G190A	M46L	7	1	7
82		2008	High prev	Eritrea	380	C			M46IM, L76LV	7	3	3

Susceptibility was predicted using the Rega resistance interpretation algorithm (V6.4.1) (http://hivdb.stanford.edu). MSM, men who have sex with men; MCTC, mother-to-child transmission; HSX, heterosexual; IDU, intravenous drug user; NNRTI, non-nucleoside reverse transcriptase inhibitor; NRTI, nucleoside reverse transcriptase inhibitor; PI, protease inhibitor.

### Phylogenetic clustering of TDR viruses

**Figure 1:**
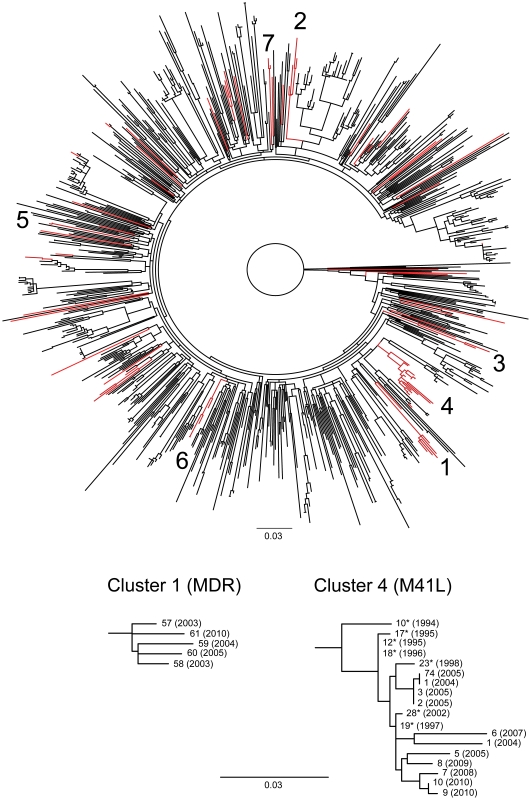
Maximum likelihood phylogenetic analysis of study subjects infected with HIV-1 of subtype B. The upper panel shows the complete tree, which includes sequences from 605 study subjects, 194 MSM diagnosed as HIV-1 infected in Stockholm 1992–2002 [17], and four subtype B and four subtype D reference sequences (www.hiv.lanl.gov). The tree was generated using partial HIV-1 pol gene sequences using PhyML and the best fitted nucleotide substitution model (i.e. GTR+I+G). Twenty-three codons with resistance mutations were removed from the alignment so that the final alignment contained 987 unambiguously aligned nucleotides. The tree is rooted using the four subtype D reference sequences. Red branches represent sequences with mutations indicative of TDR according to the WHO 2009 list of mutations for surveillance of transmitted drug resistance [20]. Numbers indicate significantly supported (aLRT>0.95) clusters of TDR sequences. The two bottom panels show detailed sub-trees for TDR clusters 1 and 4. The asterisks in the sequence identifiers in cluster 4 indicate that these sequences were obtained from the 1992–2002 dataset [17]. The scale indicates nucleotide substitutions per site.

ML phylogenetic trees were constructed for each HIV-1 subtype to investigate the relationships of viruses with TDR mutations relative to a background of viruses without such mutations. We identified nine clusters that contained two or more TDR viruses and were significantly supported with aLRT values>0.95. The characteristics of the patients involved these nine clusters are given in Tables 3 and 4. Seven of the clusters were of subtype B, one cluster was classified as CRF01_AE and finally one cluster consisted of two viruses of unclassifiable subtype. Among 42 patients with TDR who were infected in Sweden, 23 (55%) were involved in clusters. In contrast, only 5 of 33 (27%) patients infected abroad were involved in clusters (p = 0.004, Fisher exact test). Figure 1 shows a ML tree for subtype B sequences, in which the seven subtype B TDR clusters are highlighted. In this analysis we also included 194 subtype B sequences from MSM diagnosed as HIV infected in Stockholm in 1992–2002 [17]. Four of the seven subtype B clusters included sequences from the 1992–2002 dataset. The largest TDR cluster (cluster 4) consisted of 18 MSM from Stockholm with viruses that had the M41L resistance mutation (Figure 1). Two of the M41L viruses had additional mutations (T215N and M46LM, respectively). Eleven of the 18 patients in the M41L cluster were part of the present study and seven belonged to the 1992–2002 dataset. The first patient in the M41L cluster was diagnosed in 1994 [17]. Among the 11 patients diagnosed in 2003–2010, we found that seven patients had recent infections as evidenced by a documented primary HIV-1 infection (n = 2) or a negative HIV-1 antibody assay <1 year prior to diagnosis (n = 5). This includes two patients diagnosed in 2010, which shows that the M41L variant has been circulating in Stockholm between 1994 and 2010. Cluster no. 1 was the second largest cluster and consisted of all five MDR viruses, which were observed in MSM diagnosed between 2003 and 2010 (Figure 1). Remaining subtype B TDR clusters were small and contained two to four sequences.

## Discussion

In this first comprehensive study of TDR in Sweden we have prospectively investigated a representative sample of 1463 persons who were newly diagnosed with HIV-1 infection between 2003 and 2010. We found that the prevalence of TDR was relatively low, 5.6% (95% CI: 4.5%–6.9%) and stable over time. TDR was positively associated with the MSM transmission route, subtype B infection and negatively associated with CD4 cell counts.

The prevalence of TDR in Sweden was low compared to many other European countries, the U.S. as well as the pan-European SPREAD study [7]–[14]. The comparably low prevalence of TDR in Sweden in part can be explained by the fact that 32% of the study subjects were immigrants from high-prevalence countries, where access to ART sometimes has been limited. However, it should be pointed out that 12.3% of the infections occurred in Thailand, which is a high-prevalence country, but where ART has been available for a number of years and where a recent report indicated a TDR prevalence of 14% [30]. The large proportion of patients originating from and infections occurring in Thailand probably reflects the fact that Thailand is a popular travel destination for Swedish tourists and that there is significant immigration from Thailand. Another reason for the low prevalence of TDR might be that a high proportion of patients on ART in Sweden have fully suppressed virus replication with plasma HIV-1 RNA levels <50 copies per mL as shown in the Swedish national register InfCareHIV (national average 92% in 2010, http://infcare.se/hiv). The risk of sexual HIV-1 transmission from patients with “undetectable” virus levels is very low [31]–[33], which means that most transmissions occur from subjects without ongoing treatment.

The prevalence of TDR varied from 2.1% in 2006 to 7.5% in 2009, but there was no significant trend over time in the entire study population or in individual transmission routes. However, there was a non-significant increase in TDR among patients from high-endemic countries (p = 0.071). Although this non-significant trend should be interpreted with caution, it is in line with recent reports showing an increasing prevalence of TDR among sub-Saharan Africans residing in Spain [34] as well as indications of increasing levels of TDR in sub-Saharan Africa and Asia, including Thailand [30], [35].

Phylogenetic tree analyses were used to investigate clustering of sequences from the study subjects with TDR. We found that more than half (55%) of the TDR patients infected in Sweden were involved in clusters, in contrast to 27% of those reported to have been infected abroad. One cluster was large and contained 18 viruses with the M41L resistance mutation. This M41L cluster represents continued spread of a virus variant that has already has been reported in seven MSM in Stockholm [17]. The first patient in this transmission cluster was infected in 1994 (or earlier) and the last two in 2010, which shows that this virus variant has been transmitted in Stockholm over a period of at least 16 years and that the M41L mutation is very stable. Another cluster contained five viruses that were predicted to have high-level resistance to most NRTIs, NNRTIs and PIs. Clustering of TDR viruses have been reported before and has been interpreted to suggest onward transmission of TDR viruses [36], [37]. It is difficult to estimate how often onward transmission occurred in our study, but it is likely that some of the 18 patients in the M41L cluster represent onward transmission because it is improbable that one patient transmitted to all others.

As reported in some earlier studies a majority (54 of 82; 68%) of the patients with TDR had viruses with singleton resistance mutations [9], [13]. Many of these viruses with singleton mutations and also some viruses with two or more resistance mutations were predicted to be fully sensitive to all registered NRTIs, NNRTIs and PIs. Thus, the corresponding patients would be expected to respond well to standard ART. However, we cannot exclude the possibility that some patients with or without signs of TDR in bulk sequences might have resistant viruses represented as minority HIV-1 variants that might impact negatively on response to ART [38]. However, TDR may also be over-estimated because of the presence of natural sequence polymorphisms [39]. Some other limitations of the study should be mentioned. First, the study covered 44%, but not all, patients diagnosed in Sweden during the study period from January 2003 to June 2010. Second, MSM were somewhat over-represented in the study population, which may suggest that the true prevalence of TDR in Sweden may be somewhat lower than our estimate of 5.6%.

In conclusion, this study shows that the prevalence of TDR in Sweden 2003–2010 was lower than in many other European countries, but that TDR was concentrated among MSM. In this group, we also found evidence for clustering of TDR strains, which highlights the need for continued and improved measures for targeted interventions.

## Supporting Information

Table S1 Comparison of characteristics of 1327 study subjects diagnosed 2003–2009 and national Swedish HIV surveillance data.
(DOCX)
