# Supplementary material for: Low Prevalence of Transmitted Drug Resistance in Patients Newly Diagnosed with HIV-1 Infection in Sweden 2003–2010
Source: PLoS One. 2012 Mar 20;7(3):e33484. doi: 10.1371/journal.pone.0033484 (PMC3308981; doi:10.1371/journal.pone.0033484)
Supplement: Table S1 — Comparison of characteristics of 1327 study subjects diagnosed 2003–2009 and national Swedish HIV surveillance data. (DOCX) [file pone.0033484.s001.docx]

**Table S1**. Comparison of characteristics of 1327 study subjects diagnosed 2003-2009 and national Swedish HIV surveillance data.

| Characteristics | Study subjects^1^ | | All patients diagnosed in Sweden 2003 - 2009 | Coverage (%) |
| --- | --- | --- | --- | --- |
| Patients | 1327 | | 3021 | 44% |
| Sex [n (%)] |  |  |  |  |
| Male | 930 | (70%) | 1857 (61%) | 50% |
| Female | 397 | (30%) | 1164 (39%) | 34% |
| Age [n (%)] |  |  |  |  |
| 0-14 | 8 | (1%) | 99 (3%) | 8% |
| 15-24 | 94 | (7%) | 317 (11%) | 30% |
| 25-49 | 990 | (75%) | 2180 (72%) | 45% |
| 50- | 235 | (18%) | 425 (14%) | 55% |
| Year of diagnosis [n (%)] |  |  |  |  |
| 2003 | 126 | (9%) | 365 (12%) | 35% |
| 2004 | 153 | (12%) | 429 (14%) | 36% |
| 2005 | 155 | (12%) | 388 (13%) | 40% |
| 2006 | 192 | (14%) | 385 (13%) | 50% |
| 2007 | 239 | (18%) | 526 (17%) | 45% |
| 2008 | 221 | (17%) | 442 (15%) | 50% |
| 2009 | 241 | (18%) | 486 (16%) | 50% |
| Route of transmission [n (%)] |  |  |  |  |
| Intravenous drug use | 125 | (9%) | 239 (8%) | 52% |
| Homosexual/bisexual | 483 | (36%) | 743 (24%) | 65% |
| Heterosexual | 684 | (52%) | 1558 (52%) | 44% |
| Mother-to-child | 7 | (1%) |  |  |
| Other/Unknown | 28 | (2%) | 481 (16%) | 6% |
| Country/Region of infection [n (%)] | | | | |
| Sweden | 571 | (43%) | 766 (28%)^2^ | 75% |
| Europe, except Sweden | 128 | (9%) |  |  |
| Sub-Saharan Africa | 308 | (23%) |  |  |
| Asia | 195 | (15%) |  |  |
| Americas | 45 | (3%) |  |  |
| Other/Missing data | 91 | (7%) |  |  |

^1^ Comparisons were limited to 2003-2009 because study recruitment stopped in July 2010.

^2^ According to national Swedish data, which report that 766 of 2763 (28%) patients with available data were infected in Sweden
